# 2-Phenyl­imidazole dihydrogen phosphate phospho­ric acid

**DOI:** 10.1107/S1600536810004927

**Published:** 2010-02-13

**Authors:** Dao-Cheng Xia, Ji-Huan Yao

**Affiliations:** a Yuncheng University, College of Chemistry, Yuncheng 044000, People’s Republic of China

## Abstract

The crystal structure of the title compound, C_9_H_9_N_2_^+^·H_2_PO_4_^−^·H_3_PO_4_, is stabilized by N—H⋯O and O—H⋯O hydrogen-bonding inter­actions, resulting in a two-dimensional network.

## Related literature

For related structures, see: Liu *et al.* (2008 ▶); Yang *et al.* (2008 ▶); Xia *et al.* (2009 ▶).
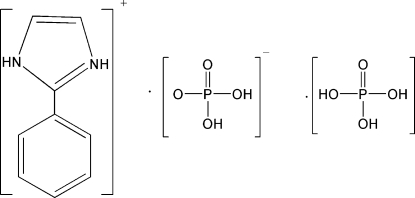


## Experimental

### 

#### Crystal data

C_9_H_9_N_2_^+^·H_2_PO_4_^−^·H_3_PO_4_
*M* *_r_* = 340.16
Monoclinic, 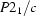

*a* = 17.1875 (12) Å
*b* = 4.7220 (3) Å
*c* = 17.7585 (14) Å
β = 99.767 (7)°
*V* = 1420.38 (17) Å^3^
*Z* = 4
Mo *K*α radiation
μ = 0.35 mm^−1^
*T* = 293 K
0.25 × 0.22 × 0.20 mm

#### Data collection

Oxford Diffraction Gemini R Ultra diffractometer
Absorption correction: multi-scan (*CrysAlis RED*; Oxford Diffraction, 2006 ▶) *T*_min_ = 0.61, *T*_max_ = 0.84
5555 measured reflections
2893 independent reflections
1549 reflections with *I* > 2.0 σ(*I*)
*R*_int_ = 0.038

#### Refinement

*R*[*F* ^2^ > 2σ(*F* ^2^)] = 0.040
*wR*(*F* ^2^) = 0.080
*S* = 0.87
2893 reflections
190 parameters
H-atom parameters constrained
Δρ_max_ = 0.27 e Å^−3^
Δρ_min_ = −0.37 e Å^−3^

### 

Data collection: *CrysAlis CCD* (Oxford Diffraction, 2006 ▶); cell refinement: *CrysAlis CCD*; data reduction: *CrysAlis RED* (Oxford Diffraction, 2006 ▶); program(s) used to solve structure: *SHELXS97* (Sheldrick, 2008 ▶); program(s) used to refine structure: *SHELXL97* (Sheldrick, 2008 ▶); molecular graphics: *SHELXTL* (Sheldrick, 2008 ▶); software used to prepare material for publication: *SHELXTL*.

## Supplementary Material

Crystal structure: contains datablocks global, I. DOI: 10.1107/S1600536810004927/pv2259sup1.cif

Structure factors: contains datablocks I. DOI: 10.1107/S1600536810004927/pv2259Isup2.hkl

Additional supplementary materials:  crystallographic information; 3D view; checkCIF report

## Figures and Tables

**Table 1:** Hydrogen-bond geometry (Å, °)

*D*—H⋯*A*	*D*—H	H⋯*A*	*D*⋯*A*	*D*—H⋯*A*
O5—H5*A*⋯O2^i^	0.82	1.91	2.563 (2)	136
O3—H3*A*⋯O2^ii^	0.82	1.76	2.546 (2)	159
O8—H8*A*⋯O6^iii^	0.82	2.01	2.553 (2)	123
N2—H2⋯O6^iii^	0.86	2.05	2.859 (3)	157
N1—H1*B*⋯O4	0.86	2.02	2.871 (3)	169
O7—H7⋯O4^iv^	0.82	1.76	2.536 (3)	158
O1—H1⋯O3^iii^	0.82	2.19	2.625 (2)	113

Symmetry codes: (i) 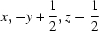; (ii) 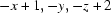; (iii) 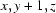; (iv) 
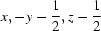
.

**Fig. 1.**
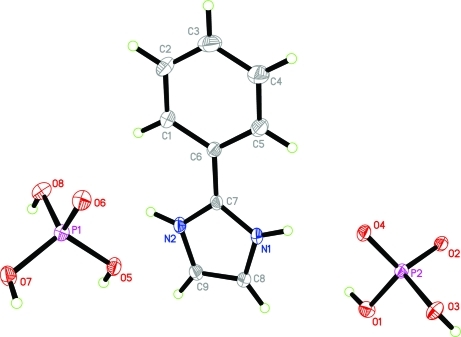
The structure of (I), showing the atomic numbering scheme. Displacement ellipsoids are drawn at the 30% probability level.

## supplementary crystallographic information

### Comment

2-Phenylimidazole is a good candidate for building supramolecular architectures (Liu *et al.*, 2008; Yang *et al.*, 2008). Continuing our research in this important field (Xia *et al.*, 2009), we now report the preparation and crystal structure of the title compound, (I).

The asymmetric unit of the title compound contains one 2-phenylimidazole cation, one dihydrogen phosphate anion and one phosphoric acid molecule (Fig. 1). The structure is stabilized by the O—H···O and N—H···O H–bonding interactions (Table 1); a rather weak interaction of the type C—H···O is also present in the structure.

### Experimental

A mixture of 2-phenylimidazole (0.5 mmol), phosphoric acid (1 mmol) and H_2_O (30 mmol) was mixed. After two weeks, colorless crystals of (I) were yielded at room temperature (18% yield).

### Refinement

All H atoms on C and N atoms were positioned geometrically with distances O—H, N—H and C—H = 0.82, 0.86 and 0.93 Å, respectively, and were refined in riding mode, with U_iso_(H) = 1.5U_eq_(O) and 1.2U_eq_(C/N).

### Crystal data

**Table:**

C_9_H_9_N_2_^+^·H_2_PO_4_^−^·H_3_PO_4_	*F*(000) = 704
*M**_r_* = 340.16	*D*_x_ = 1.591 Mg m^−^^3^
Monoclinic, *P*2_1_/*c*	Mo *K*α radiation, λ = 0.71073 Å
Hall symbol: -P 2ybc	Cell parameters from 2893 reflections
*a* = 17.1875 (12) Å	θ = 2.3–26.4°
*b* = 4.7220 (3) Å	µ = 0.35 mm^−^^1^
*c* = 17.7585 (14) Å	*T* = 293 K
β = 99.767 (7)°	Block, colorless
*V* = 1420.38 (17) Å^3^	0.25 × 0.22 × 0.20 mm
*Z* = 4	

### Data collection

**Table:**

Oxford Diffraction Gemini R Ultra diffractometer	2893 independent reflections
Radiation source: fine-focus sealed tube	1549 reflections with *I* > 2.0 σ(*I*)
graphite	*R*_int_ = 0.038
Detector resolution: 10.0 pixels mm^-1^	θ_max_ = 26.4°, θ_min_ = 2.3°
ω scan	*h* = −21→21
Absorption correction: multi-scan (*CrysAlis RED*; Oxford Diffraction, 2006)	*k* = −5→4
*T*_min_ = 0.61, *T*_max_ = 0.84	*l* = −13→22
5555 measured reflections	

### Refinement

**Table:**

Refinement on *F*^2^	Primary atom site location: structure-invariant direct methods
Least-squares matrix: full	Secondary atom site location: difference Fourier map
*R*[*F*^2^ > 2σ(*F*^2^)] = 0.040	Hydrogen site location: inferred from neighbouring sites
*wR*(*F*^2^) = 0.080	H-atom parameters constrained
*S* = 0.87	*w* = 1/[σ^2^(*F*_o_^2^) + (0.0302*P*)^2^] where *P* = (*F*_o_^2^ + 2*F*_c_^2^)/3
2893 reflections	(Δ/σ)_max_ < 0.001
190 parameters	Δρ_max_ = 0.27 e Å^−^^3^
0 restraints	Δρ_min_ = −0.37 e Å^−^^3^

### Special details

**Table:**

Geometry. All esds (except the esd in the dihedral angle between two l.s. planes) are estimated using the full covariance matrix. The cell esds are taken into account individually in the estimation of esds in distances, angles and torsion angles; correlations between esds in cell parameters are only used when they are defined by crystal symmetry. An approximate (isotropic) treatment of cell esds is used for estimating esds involving l.s. planes.
Refinement. Refinement of *F*^2^ against ALL reflections. The weighted *R*-factor wR and goodness of fit *S* are based on *F*^2^, conventional *R*-factors *R* are based on *F*, with *F* set to zero for negative *F*^2^. The threshold expression of *F*^2^ > σ(*F*^2^) is used only for calculating *R*-factors(gt) etc. and is not relevant to the choice of reflections for refinement. *R*-factors based on *F*^2^ are statistically about twice as large as those based on *F*, and *R*- factors based on ALL data will be even larger.

### Fractional atomic coordinates and isotropic or equivalent isotropic displacement parameters (Å^2^)

**Table:**

	*x*	*y*	*z*	*U*_iso_*/*U*_eq_	
P1	0.21882 (4)	−0.03732 (14)	0.43687 (4)	0.0302 (2)	
P2	0.42236 (4)	0.04439 (15)	0.89806 (4)	0.0299 (2)	
O4	0.35030 (10)	−0.0447 (4)	0.84416 (10)	0.0393 (5)	
O5	0.28575 (10)	0.0703 (4)	0.50061 (9)	0.0381 (5)	
H5A	0.3047	0.2162	0.4864	0.057*	
O3	0.48015 (11)	−0.2115 (3)	0.91299 (11)	0.0381 (5)	
H3A	0.5199	−0.1635	0.9425	0.057*	
O6	0.18630 (11)	−0.3002 (3)	0.46455 (11)	0.0388 (5)	
O2	0.40773 (10)	0.1605 (4)	0.97411 (10)	0.0380 (5)	
O7	0.25178 (11)	−0.0717 (4)	0.36199 (10)	0.0428 (5)	
H7	0.2877	−0.1882	0.3682	0.064*	
O1	0.46827 (11)	0.2710 (4)	0.85825 (11)	0.0399 (5)	
H1	0.4399	0.4091	0.8466	0.060*	
O8	0.15458 (10)	0.1928 (4)	0.41974 (11)	0.0405 (5)	
H8A	0.1738	0.3365	0.4045	0.061*	
N2	0.26383 (14)	0.5520 (5)	0.61475 (12)	0.0382 (6)	
H2	0.2309	0.6152	0.5765	0.046*	
N1	0.31292 (13)	0.3278 (5)	0.71534 (12)	0.0395 (6)	
H1B	0.3176	0.2181	0.7545	0.047*	
C5	0.16191 (18)	0.0460 (7)	0.72484 (19)	0.0548 (9)	
H5	0.2037	0.0178	0.7649	0.066*	
C8	0.37239 (17)	0.4892 (6)	0.69554 (17)	0.0443 (8)	
H8	0.4242	0.4991	0.7212	0.053*	
C6	0.17162 (16)	0.2248 (6)	0.66598 (16)	0.0361 (7)	
C3	0.0300 (2)	−0.0510 (8)	0.6673 (2)	0.0657 (10)	
H3	−0.0174	−0.1454	0.6676	0.079*	
C9	0.34158 (17)	0.6299 (6)	0.63224 (17)	0.0429 (8)	
H9	0.3679	0.7565	0.6052	0.051*	
C2	0.03804 (19)	0.1260 (9)	0.6094 (2)	0.0719 (11)	
H2A	−0.0043	0.1531	0.5699	0.086*	
C7	0.24664 (16)	0.3649 (6)	0.66528 (15)	0.0332 (7)	
C1	0.10825 (19)	0.2679 (8)	0.60773 (18)	0.0604 (10)	
H1A	0.1128	0.3911	0.5678	0.072*	
C4	0.0923 (2)	−0.0905 (8)	0.7256 (2)	0.0691 (10)	
H4	0.0870	−0.2109	0.7658	0.083*	

### Atomic displacement parameters (Å^2^)

**Table:**

	*U*^11^	*U*^22^	*U*^33^	*U*^12^	*U*^13^	*U*^23^
P1	0.0327 (4)	0.0264 (4)	0.0305 (4)	−0.0009 (3)	0.0027 (3)	−0.0005 (3)
P2	0.0291 (4)	0.0297 (4)	0.0301 (4)	0.0019 (3)	0.0027 (3)	0.0022 (3)
O4	0.0320 (10)	0.0512 (12)	0.0313 (11)	−0.0061 (9)	−0.0042 (9)	0.0072 (9)
O5	0.0402 (11)	0.0425 (11)	0.0293 (11)	−0.0111 (9)	−0.0012 (9)	0.0036 (9)
O3	0.0348 (11)	0.0290 (10)	0.0457 (13)	0.0045 (8)	−0.0063 (9)	−0.0022 (9)
O6	0.0451 (12)	0.0271 (10)	0.0430 (13)	−0.0067 (9)	0.0036 (10)	−0.0010 (9)
O2	0.0331 (11)	0.0520 (12)	0.0283 (11)	0.0102 (9)	0.0033 (9)	−0.0007 (9)
O7	0.0525 (13)	0.0445 (12)	0.0315 (12)	0.0114 (10)	0.0077 (9)	0.0030 (9)
O1	0.0448 (12)	0.0311 (10)	0.0461 (13)	0.0029 (9)	0.0136 (10)	0.0066 (9)
O8	0.0350 (12)	0.0289 (10)	0.0547 (15)	0.0002 (9)	−0.0011 (10)	−0.0008 (9)
N2	0.0431 (15)	0.0443 (14)	0.0263 (14)	0.0063 (12)	0.0031 (11)	0.0070 (12)
N1	0.0407 (16)	0.0522 (15)	0.0246 (14)	−0.0007 (12)	0.0023 (12)	0.0090 (11)
C5	0.0418 (19)	0.065 (2)	0.054 (2)	−0.0073 (17)	−0.0042 (16)	0.0145 (18)
C8	0.0353 (17)	0.061 (2)	0.0354 (18)	−0.0041 (15)	0.0028 (14)	0.0083 (16)
C6	0.0346 (18)	0.0438 (17)	0.0296 (19)	0.0066 (14)	0.0049 (14)	−0.0059 (14)
C3	0.042 (2)	0.079 (3)	0.077 (3)	−0.0129 (19)	0.013 (2)	−0.012 (2)
C9	0.0433 (19)	0.0520 (19)	0.0337 (19)	−0.0065 (16)	0.0073 (15)	0.0051 (15)
C2	0.041 (2)	0.117 (3)	0.051 (3)	−0.001 (2)	−0.0093 (18)	−0.004 (2)
C7	0.0368 (17)	0.0394 (16)	0.0234 (16)	0.0082 (14)	0.0052 (14)	−0.0019 (13)
C1	0.041 (2)	0.097 (3)	0.039 (2)	−0.0023 (18)	−0.0026 (17)	0.0111 (18)
C4	0.051 (2)	0.081 (3)	0.074 (3)	−0.0155 (19)	0.007 (2)	0.018 (2)

### Geometric parameters (Å, °)

**Table:**

P1—O6	1.4790 (18)	N1—C8	1.368 (3)
P1—O7	1.5400 (19)	N1—H1B	0.8600
P1—O8	1.5421 (18)	C5—C4	1.361 (4)
P1—O5	1.5559 (17)	C5—C6	1.376 (4)
P2—O4	1.4916 (18)	C5—H5	0.9300
P2—O2	1.5175 (19)	C8—C9	1.335 (4)
P2—O3	1.5579 (18)	C8—H8	0.9300
P2—O1	1.568 (2)	C6—C1	1.384 (4)
O5—H5A	0.8200	C6—C7	1.451 (4)
O3—H3A	0.8200	C3—C2	1.350 (5)
O7—H7	0.8200	C3—C4	1.370 (4)
O1—H1	0.8200	C3—H3	0.9300
O8—H8A	0.8200	C9—H9	0.9300
N2—C7	1.328 (3)	C2—C1	1.385 (5)
N2—C9	1.370 (3)	C2—H2A	0.9300
N2—H2	0.8600	C1—H1A	0.9300
N1—C7	1.332 (3)	C4—H4	0.9300
			
O6—P1—O7	114.43 (11)	C6—C5—H5	119.4
O6—P1—O8	111.01 (11)	C9—C8—N1	106.7 (3)
O7—P1—O8	105.07 (11)	C9—C8—H8	126.7
O6—P1—O5	107.86 (10)	N1—C8—H8	126.7
O7—P1—O5	109.12 (10)	C5—C6—C1	118.5 (3)
O8—P1—O5	109.25 (10)	C5—C6—C7	120.6 (3)
O4—P2—O2	115.35 (11)	C1—C6—C7	120.9 (3)
O4—P2—O3	109.06 (11)	C2—C3—C4	119.5 (3)
O2—P2—O3	109.01 (10)	C2—C3—H3	120.3
O4—P2—O1	109.26 (11)	C4—C3—H3	120.3
O2—P2—O1	109.08 (11)	C8—C9—N2	106.8 (3)
O3—P2—O1	104.53 (11)	C8—C9—H9	126.6
P1—O5—H5A	109.5	N2—C9—H9	126.6
P2—O3—H3A	109.5	C3—C2—C1	121.2 (3)
P1—O7—H7	109.5	C3—C2—H2A	119.4
P2—O1—H1	109.5	C1—C2—H2A	119.4
P1—O8—H8A	109.5	N2—C7—N1	106.0 (2)
C7—N2—C9	110.2 (2)	N2—C7—C6	127.5 (2)
C7—N2—H2	124.9	N1—C7—C6	126.5 (3)
C9—N2—H2	124.9	C6—C1—C2	119.4 (3)
C7—N1—C8	110.3 (2)	C6—C1—H1A	120.3
C7—N1—H1B	124.9	C2—C1—H1A	120.3
C8—N1—H1B	124.9	C5—C4—C3	120.3 (3)
C4—C5—C6	121.2 (3)	C5—C4—H4	119.9
C4—C5—H5	119.4	C3—C4—H4	119.9

### Hydrogen-bond geometry (Å, °)

**Table:**

*D*—H···*A*	*D*—H	H···*A*	*D*···*A*	*D*—H···*A*
O5—H5A···O2^i^	0.82	1.91	2.563 (2)	136
O3—H3A···O2^ii^	0.82	1.76	2.546 (2)	159
O8—H8A···O6^iii^	0.82	2.01	2.553 (2)	123
N2—H2···O6^iii^	0.86	2.05	2.859 (3)	157
N1—H1B···O4	0.86	2.02	2.871 (3)	169
O7—H7···O4^iv^	0.82	1.76	2.536 (3)	158
O1—H1···O3^iii^	0.82	2.19	2.625 (2)	113
C9—H9···O5^iii^	0.93	2.60	3.154 (3)	119

Symmetry codes: (i) *x*, −*y*+1/2, *z*−1/2; (ii) −*x*+1, −*y*, −*z*+2; (iii) *x*, *y*+1, *z*; (iv) *x*, −*y*−1/2, *z*−1/2.
